# Proteolytic α-Synuclein Cleavage in Health and Disease

**DOI:** 10.3390/ijms22115450

**Published:** 2021-05-21

**Authors:** Alexandra Bluhm, Sarah Schrempel, Stephan von Hörsten, Anja Schulze, Steffen Roßner

**Affiliations:** 1 Flechsig Institute for Brain Research, University of Leipzig, 04103 Leipzig, Germany; alexandra.bluhm@medizin.uni-leipzig.de (A.B.); sarah.schrempel@medizin.uni-leipzig.de (S.S.); 2 Department for Experimental Therapy, University Clinics Erlangen and Preclinical Experimental Center, Friedrich-Alexander-Universität Erlangen-Nürnberg, 91054 Erlangen, Germany; stephan.v.hoersten@fau.de; 3 Department of Molecular Drug Design and Target Validation, Fraunhofer Institute for Cell Therapy and Immunology, 06120 Halle/Saale, Germany; anja.schulze@izi.fraunhofer.de

**Keywords:** α-synuclein, post-translational modification, proteolysis, Parkinson’s disease, dementia with Lewy bodies, substantia nigra, animal models

## Abstract

In Parkinson’s disease, aggregates of α-synuclein within Lewy bodies and Lewy neurites represent neuropathological hallmarks. However, the cellular and molecular mechanisms triggering oligomeric and fibrillary α-synuclein aggregation are not fully understood. Recent evidence indicates that oxidative stress induced by metal ions and post-translational modifications such as phosphorylation, ubiquitination, nitration, glycation, and SUMOylation affect α-synuclein conformation along with its aggregation propensity and neurotoxic profiles. In addition, proteolytic cleavage of α-synuclein by specific proteases results in the formation of a broad spectrum of fragments with consecutively altered and not fully understood physiological and/or pathological properties. In the present review, we summarize the current knowledge on proteolytical α-synuclein cleavage by neurosin, calpain-1, cathepsin D, and matrix metalloproteinase-3 in health and disease. We also shed light on the contribution of the same enzymes to proteolytical processing of pathogenic proteins in Alzheimer’s disease and report potential cross-disease mechanisms of pathogenic protein aggregation.

## 1. Introduction

Parkinson’s disease (PD; *Paralysis agitans*) is a progressive neurodegenerative disorder. After Alzheimer’s disease (AD), it represents the second most frequent neurodegenerative disease entity with the vast majority of cases being idiopathic [1,2]. On the neuronal level, PD is characterized by the degeneration of dopaminergic substantia nigra (SN) neurons, resulting in dopaminergic striatal under-supply and hypofunction [3,4,5]. Motor findings become clinically manifest only after a loss of 50% of SN neurons associated with approximately 80% of striatal dopamine depletion [3]. During disease progression, the extent of dopaminergic hypoactivity correlates to the severity of clinical symptoms [6,7,8].

PD is not only a neurodegenerative disorder affecting dopaminergic neurons, but it can also be considered as a protein aggregation disorder. In that respect, it shares similarities to AD and Huntington disease that are also characterized by the accumulation of pathogenic protein species. In PD, such aggregates appear as Lewy bodies and Lewy neurites being mainly composed of aggregated α-synuclein [9,10,11,12]. α-Synuclein is a member of a family of proteins that also includes β-synuclein [13,14] and γ-synuclein [15] and was termed based on its primary localization to the presynaptic compartment and to the nucleus [16]. Structurally, α-synuclein consists of 140 amino acids (aa) with an amphipathic N-terminal region (aa 1–60), a central hydrophobic domain involved in protein aggregation (non-Aβ component (NAC) region; aa 61–95), and a highly acidic C-terminus (aa 96–140) [17]. Under physiological conditions, it exists as a soluble, natively unfolded protein and in a membrane-bound α-helical structure important for SNARE-complex formation and vesicle fusion [18,19]. However, in the course of PD, α-synuclein conformation is altered to form β-sheet oligomers, which may be converted into amyloid fibrils and give rise to Lewy bodies and Lewy neurites [9,20]. The importance of different α-synuclein strains for disease heterogeneity, specific characteristics of different synucleinopathies, and phenotypes in Lewy body dementia has been established recently [21,22,23]. The trigger for α-synuclein aggregation is not known but could involve oxidative stress induced by Cu^2+^ or Fe^3+^ [24,25,26].

In familial PD, a number of α-synuclein mutations such as A30P, E46K, and A53T have been identified and were shown to affect fibril formation and structure [27,28,29]. However, most PD cases are sporadic and do not have a genetic predisposition. Therefore, we here focus on the processing of wild-type α-synuclein. The full-length α-synuclein may undergo a magnitude of post-translational modifications including but not limited to phosphorylation, ubiquitination, nitration, glycation, SUMOylation, and proteolytical truncation [30,31,32]. Such post-translational modifications of α-synuclein alter overall protein hydrophobicity, and binding affinities to other proteins and lipids and have been extensively studied with respect to the regulation of their physiological functions as well as to their contribution to pathological processes in PD [19,32]. The most prominent disease-related modification is α-synuclein phosphorylation at serine 129 [30,33,34,35,36], which is considered an early peripheral diagnostic marker [37,38,39,40,41,42].

**Figure 1:**
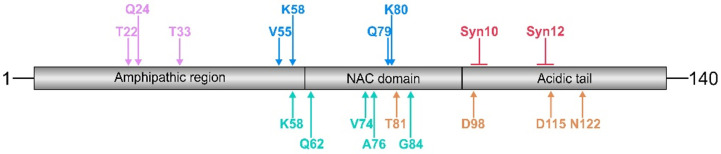
Schematic presentation of cleavage sites within the α-synuclein molecule generated by trypsin (purple), MMP-3 (blue), calpain-1 (green), cathepsin D (red), and neurosin (orange). Cathepsin D induces the formation of 10 kDa and 12 kDa α-synuclein fragments (Syn10 and Syn12, respectively). Note that cleavage sites for a given protease cluster in distinct α-synuclein domains, such as the N-terminus (trypsin), the NAC domain (MMP-3 and calpain-1), and the C-terminus (cathepsin D and neurosin). The numbers above the arrows indicate the position of the free N-terminal amino acid generated. For further details, subcellular localization, and references, see Table 1.

Generally, two types of proteolytic pathways are involved in α-synuclein degradation: the cytosolic ubiquitin/proteasome pathway and, more importantly, the autophagic/lysosomal pathways, including macro-autophagy and chaperone-mediated autophagy [43,44,45,46]. Nonetheless, proteolytic cleavage of α-synuclein remains a significant but yet underestimated modification, which may result in a loss of function of the maternal full-length protein and in additional physiological and/or pathological functions of the N- and C-terminal fragments generated. For example, trypsin digestion of α-synuclein resulted in cleavage between aa 21 and 22, between aa 23 and 24, and between aa 32 and 33 [47] (Figure 1 and Table 1). Furthermore, plasmin was demonstrated to degrade monomeric as well as aggregated extracellular α-synuclein in a dose- and time-dependent manner [48]. This cleavage mainly occurred at the N-terminal region and within the NAC domain of α-synuclein and inhibited the transmission of extracellular α-synuclein into the neighboring cells [48]. Intriguingly, plasmin was also shown to degrade Aβ peptides that typically accumulate in AD brains [49,50], and trypsin was reported to interact with amyloid precursor protein (APP) in senile plaques in AD [51].

Here, we focus on α-synuclein cleavage by neurosin, calpain-1, cathepsin D, and matrix metalloproteinases (MMPs), which were extensively studied as α-synuclein degrading enzymes. However, their contributions to the maintenance of physiological functions and initiation of pathogenic aspects of synucleinopathies need to be validated. For example, truncated α-synuclein fragments have been detected in brains of healthy individuals but also in the Lewy bodies of PD patients [52,53,54,55]. Additionally, these fragments have been linked to neurotoxicity, they display increased aggregation propensity and may act as seeds for aggregation of yet unmodified, native full-length α-synuclein [53,56,57,58,59,60]. This scenario is similar to the processing of the APP by the β-secretase BACE1, which is also active under physiological conditions. However, when over-activated or when degradation of the resulting Aβ peptides is compromised, BACE1 initiates amyloid pathology in AD. Intriguingly, Aβ truncation [61,62] and other post-translational modifications such as phosphorylation [63,64,65], nitration [66], and pyroglutamination [67,68] also generate seeds for the (co)-aggregation of maternal Aβ peptides. Thus, defined proteolytic protein degradation might be a general mechanism shared between different clinical entities characterized by pathological protein aggregation. There is also solid evidence reported below that supports a role of α-synuclein-cleaving proteases in the processing of APP and/or Aβ peptides in AD. Although there are no genetic data to prove such an implication of these enzymes in AD pathogenesis, they may contribute to pathological protein processing, which in turn is appearing downstream of events such as oxidative stress, neuroinflammation, and neurodegeneration. We here highlight the specific fragmentation processes of α-synuclein induced by defined proteases and their potential contribution to pathogenic mechanisms underlying protein aggregation in human synucleinopathies.

## 2. α-Synuclein Cleavage by Neurosin

Neurosin, *alias* Kallikrein-6 (KLK-6), Zyme, and Protease M, is a trypsin-like serine protease that was cloned in 1997 and found to be preferentially expressed in brain [69]. It is a 244 aa protein that shares homology with trypsinogen I and II and was initially believed to act in the interstitial space of the brain [70]. The first immunohistochemical demonstration of neurosin in human brain tissue revealed labeling of glial nuclei and of neuronal nucleoli, cytoplasm, and neurites [70]. The neuronal and glial neurosin expression was validated by in situ hybridization. In SN of control subjects, some neuromelanin-positive neurons were neurosin-immunoreactive, whereas in PD, neurosin was associated with both neuromelanin-positive neurons and with Lewy bodies [70]. In human brain tissue from PD and Multiple System Atrophy (MSA) patients, neurosin was detected by immunohistochemical labelings in the core of Lewy bodies and in glial cytoplasmic inclusions in MSA [71].

Neurosin was subsequently shown to co-localize with α-synuclein in mouse brain neurons and to be released from mitochondria into the cytosol upon cellular stress [71]. For proteolytic cleavage, the substrate and active form of the enzyme need to co-localize in the respective subcellular compartments. Given the predominant localization of α-synuclein to the nucleus and to synapses, and the reported mitochondrial neurosin localization, there is an obvious issue for physiological processing. Biochemical assays revealed α-synuclein cleavage by neurosin, which could be blocked dose-dependently by KLK inhibitor [71]. In addition, the down-regulation of neurosin expression in cultured cells by siRNA caused intracellular accumulation of α-synuclein. Tatebe et al. [72] demonstrated that neurosin transfected into HeLa cells is mostly localized to the ER, but rarely to mitochondria and lysosomes. In addition, there is significant neurosin secretion into the culture medium. Intriguingly, gelatin zymography revealed the absence of enzymatic activity from intracellular extracts, whereas extracellular neurosin showed gelatinolytic activity. This would preclude intracellular α-synuclein cleavage by neurosin but favors the extracellular clearance of α-synuclein. Indeed, after the co-expression of neurosin and α-synuclein, no α-synuclein cleavage occurred within cells, whereas extracellular α-synuclein was degraded by secreted neurosin [72].

Kasai et al. [73] determined the exact sites of α-synuclein cleavage by neurosin. Upon degradation of α-synuclein by neurosin, one major and three additional cleavage products were detected by Western blot analysis. The identity of these fragments was revealed subsequently by liquid chromatography-ion trap mass spectrometry. The prevalent cleavage site was identified within the NAC domain at K80/T81 (Figure 1), indicating that this specific cleavage accounts for reduced α-synuclein aggregation reported by Iwata et al. [71]. However, the positions of the other cleavage sites (K97/D98, E114/D115, and D121/N122) are in the C-terminal part of the α-synuclein molecule (Figure 1 and Table 1) and therefore likely to increase α-synuclein aggregation. Overall, α-synuclein cleavage by neurosin was shown to prevent its aggregation by reducing the concentration of monomer and by generating fragments that themselves inhibit polymerization [71]. Thus, the exact balance between the generation of different fragments by neurosin and other proteases, as well as additional post-translational modifications is decisive for the net outcome on the aggregation process. This is exemplified by a remarkable resistance of pSer129-α-synuclein against degradation by neurosin [73].

The implication of neurosin in proteolytic α-synuclein processing prompted the idea of using neurosin as a peripheral marker for synucleinopathies including PD and dementia with Lewy bodies (DLB). In a well-characterized human cohort, small but statistically significant reductions in PD and DLB cerebrospinal fluid (CSF) neurosin and α-synuclein concentrations were reported when compared to the control group [74]. Interestingly, AD patients demonstrated significantly increased CSF α-synuclein but similar neurosin levels compared to non-demented controls. The authors concluded that altered CSF levels of α-synuclein and neurosin in patients with synucleinopathy versus AD mirror disease-specific neuropathological mechanisms that might be useful for the development of biomarkers specific for synucleinopathies [74].

Together, the experimental data on proteolytic α-synuclein processing by neurosin reviewed above indicate that neurosin deficiency, its compromised secretion, or inhibition may result in reduced α-synuclein clearance and in the formation of oligomeric and fibrillar α-synuclein aggregates in disease-related conditions. Thorough quantitative immunohistochemical and Western blotting analyses revealed significant reductions in cortical neurosin levels in human DLB subjects and in α-synuclein transgenic mice [75]. The authors showed that neurosin degraded both monomeric and oligomeric α-synuclein and that fragments generated by neurosin were unharmful to neurons, which is consistent with predominant cleavage within the NAC domain [73]. Using a lentiviral-driven expression system for neurosin, reduced accumulation of wild-type α-synuclein and reduced α-synuclein-associated toxicity were observed in neuronal cell cultures [75]. In transgenic mice expressing human wild-type α-synuclein, lentiviral neurosin delivery markedly reduced α-synuclein accumulation, whereas the delivery of neurosin siRNA exacerbated α-synuclein aggregation [75]. Similar protective effects of neurosin in this experimental setup were seen on neuronal integrity, synapse density, and gliosis. All effects reported were specific for wild-type α-synuclein and were not observed in a transgenic mouse model expressing mutant A53T α-synuclein [75].

The same group also investigated the ability of a modified, systemically delivered neurosin to reduce the levels of α-synuclein in oligodendrocytes and reduce the cell-to-cell spread of α-synuclein to glial cells in a mouse model of MSA (MBP-α-syn [76]). They demonstrated that peripheral administration of the neurosin-apoB vector to MBP-α-synuclein mice resulted in the accumulation of neurosin-apoB in the CNS, reduced accumulation of α-synuclein in oligodendrocytes and astrocytes, improved myelin sheath formation in the corpus callosum, and behavioral improvements [76].

In addition, both recombinant and naturally secreted KLK6 (*alias* neurosin) were shown to readily cleave α-synuclein fibrils, which is likely to protect from prion-like cell-to-cell propagation [77]. These authors also generated adenoviral vectors for KLK6 delivery and demonstrated the reduction of extracellular α-synuclein by neuronally secreted KLK6. Moreover, KLK6 cleaves pro-MMP-2 and pro-ADAMTS19 to generate enzymatically active forms that could contribute to α-synuclein degradation in a cascade-like manner [77]. On the other hand, KLK6 deficiency did not affect the concentrations of intracellular or extracellular α-synuclein and its pathological accumulation [78], indicating that other proteases can compensate for the absence of KLK6. In MSA, the KLK6 protein level was elevated in putamen, but its expression level did not correlate with α-synuclein load [79].

Neurosin was also reported to play a role in AD. In this condition, the immunohistochemical neurosin labeling of neurons was reduced, and neurosin immunoreactivity was re-distributed to neurofibrillary tangle-like structures of pyramidal neurons and found to be associated with amyloid plaques [70]. The reduced neurosin expression was validated by RT-PCR [70]. The serine protease activity of neurosin may contribute to proteolytical processing of the neuronal APP695 isoform that lacks the Kunitz-type serine protease inhibitor (KPI) domain present in longer APP751 and APP770 isoforms. Thus, reduced expression or re-distribution of neurosin may result in altered APP processing, in particular of the neuronal APP695 isoform lacking the KPI domain. Indeed, the co-expression of Zyme (*alias* neurosin, *alias* KLK-6) with APP695, but not of APP751, resulted in an increased formation of Aβ peptides and its oligomeric aggregates [80]. In brains of rhesus monkeys and AD patients, Zyme was reported to be associated with cortical microvessels [80]. Moreover, a 50% reduction of KLK6 was reported in brain tissue extracts from AD patients and a concomitant 3-fold and 10-fold increase in CSF and whole blood, respectively. The same group subsequently demonstrated proteolysis of APP by KLK6 and determined the cleavage sites at the N-terminal end of the protein [81].

## 3. α-Synuclein Cleavage by Calpain–1

The predominant localization of α-synuclein to synapses suggests that membrane-associated proteases such as the calcium-activated neutral protease calpain-1 may contribute to α-synuclein degradation. Calpain-1 exhibits a broad spectrum endopeptidase-like specificity and belongs to the peptidase family C2. Its activity has been implicated in physiological pathways and in disease-related processes including stroke and AD [82,83,84,85]. Mishizen-Eberz et al. [86] analyzed the ability of calpain-1 to cleave α-synuclein in vitro by using immunoblot detection and mass spectrometric identification of the generated fragments. They detected four main fragments that were recognized by antibodies raised against different epitopes along the α-synuclein molecule. Mass spectrometry revealed the exact cleavage sites after aa 57, 73, 75, and 83 (Figure 1 and Table 1). These cleavage sites cluster within or close to the NAC domain important for α-synuclein fibrillization and, therefore, result in the formation of fragments with reduced aggregation propensity. Interestingly, these fragments were generated from soluble wild-type α-synuclein as substrate, but not from fibrillar α-synuclein. Instead, fibrillized wild-type and mutant A53T α-synuclein were predominantly cleaved within the C-terminus after aa 114 and aa 122, indicating a differential contribution of calpain-1 to α-synuclein processing under physiological and pathological conditions, respectively [86].

However, these results differ somewhat from those obtained by Dufty et al. [87]. When these authors digested α-synuclein with calpain-1 and analyzed fragments generated by N-terminal sequencing, they did not detect the major cleavage sites within the NAC domain, but they did observe cleavage after aa 9 and aa 122. Since a similar cleavage pattern was observed by Mishizen-Eberz et al. [86] for aggregated α-synuclein, it is plausible that both studies used different forms of pre-aggregated α-synuclein. Consistently, calpain-1 cleavage of α-synuclein outside the NAC domain increased aggregation and resulted in β-sheet configuration [87]. Using antibodies raised against the neo-epitopes 10–18 and 117–122, the presence of these α-synuclein fragments was demonstrated in brains of transgenic mice with synucleinopathy and in Lewy bodies and Lewy neurites in post mortem brain tissue of PD and DLB patients [87]. In MSA, the levels of calpain-1 protein and enzymatic activity were elevated in putamen and cerebellar white matter but did not correlate with α-synuclein load [79]. Novel calpain inhibitors were shown to exert disease-modifying activity and reduction of α-synuclein deposition in transgenic models of PD and DLB [88].

In addition, in vitro quantitative structure–activity relationship analysis using newly determined cleavage sites and catalytic efficiencies of an oligopeptide array identified α-synuclein cleavage between aa 61 and 62 by calpain-1 [89] (Figure 1).

Calpain-1 may also be involved in the formation and secretion of Aβ in AD [90,91]. A calpain-specific inhibitor induced a specific increase in secreted Aβ42 relative to total secreted Aβ, while a general proteasome-specific inhibitor did not show such an effect [91]. The authors concluded that the Aβ42 secretion ratio is modulated by the calpain¬calpastatin system and may open new avenues for specifically targeting Aβ42 secretion. Calpain-1 inhibition also reduced Aβ42-induced death of cortical neurons in a proteolytic pathway involving p25 activation [92], decreased Aβ levels, and preserved memory in a transgenic AD model in vivo [93]. Under conditions of disturbed calcium homeostasis, calpains might become abnormally activated and contribute to aberrant proteolytic protein processing (for a review, see [94]). For example, calpain activation was shown to promote BACE1 expression and to increase amyloid plaque formation in a transgenic AD model [95]. In human AD brain, the up-regulation of calpain activity was shown to precede tau phosphorylation and loss of synaptic proteins [96].

## 4. α-Synuclein Cleavage by Cathepsin D

Cathepsin D is a lysosomal aspartyl protease important for non-specific degradation and recycling of long-lived proteins. It has been implicated in the pathogenesis of various neurodegenerative and lysosomal storage diseases [97], but it also plays a role in postnatal tissue homeostasis and remodeling [98]. In a cellular model of Fe^2+^-induced α-synuclein oligomerization, the formation of 10 kDa and 12 kDa α-synuclein fragments (Syn10 and Syn12, respectively) was shown [99]. These truncated Syn10 and Syn12 fragments were not detected by antibodies binding to the α-synuclein C-terminus; i.e., to epitopes between aa 115 and 140, but the Syn12 fragment was recognized by antibodies binding to the epitopes 98–115 and 111–131 [99]. This indicates C-terminal cleavage of α-synuclein to obtain the Syn12 fragment (Figure 1 and Table 1). Furthermore, Syn10 fragments were identified with the Syn1 antibody binding to aa 91 to 99 but not with an antibody directed against aa 98 to 115. Thus, the Syn10 fragment is most likely generated by α-synuclein cleavage closer toward the NAC domain (Figure 1 and Table 1). This is consistent with the preservation of the NAC domain important for α-synuclein aggregation and was experimentally shown by Western blot analysis of aggregated α-synuclein [99]. The authors subsequently demonstrated that in the Fe^2+^-induced oligomerization model used, both Fe^2+^ and α-synuclein induced cathepsin D expression in an additive and specific manner, since this effect was not observed for calpain-1 and caspase-3. The contribution of cathepsin D to Syn10 and Syn12 formation was validated by interfering with cathepsin D maturation and by inhibition of its enzymatic activity with the use of NH_4_Cl and Pepstatin A treatment, respectively [99]. These treatments also reduced oligomer formation, which is consistent with the accelerated aggregation of C-terminally truncated α-synuclein variants. The role of cathepsin D as the main lysosomal enzyme for the generation of C-terminally truncated α-synuclein variants was substantiated (i) in enzymatic assays using α-synuclein and purified cathepsin D and (ii) in cathepsin D knock-down experiments using cells overexpressing wild-type α-synuclein [100]. Intriguingly, cathepsin D expression levels correlated with the formation of C-terminally truncated α-synuclein. Furthermore, α-synuclein was shown to be resistant to degradation by lysosomes with reduced cathepsin D activity [100], the whole process being pH dependent [101]. In a mouse model with cathepsin D deficiency, robust accumulation of endogenous α-synuclein was observed [102]. In cathepsin D knock-out mice, levels of soluble endogenous α-synuclein were reduced, but its insoluble oligomeric forms were elevated [103], which is consistent with a role of α-synuclein cleavage by cathepsin D in the prevention of α-synuclein oligomerization. Vice versa, cathepsin D overexpression reduced α-synuclein aggregation and protected from α-synuclein overexpression-induced cell death in vitro [102]. Similar effects were observed in the *C. elegans* model, pointing toward a general, cross-species pathway. The specificity of cathepsin D in this process was proven by comparative analyses with enzymatically inactive cathepsin D and with cathepsin B and L, neither of them being efficient in α-synuclein degradation or in neuroprotection [102]. The overexpression of cathepsin D in dopaminergic cell cultures induced extensive proteolysis of α-synuclein in a dose-dependent fashion [103]. Furthermore, the formation of intraneuronal α-synuclein inclusions, axonal swellings, and neurite pathology were observed in brains of human and sheep with cathepsin D deficiency [103]. In MSA, cathepsin D enzymatic activity was found to be elevated in pons and cerebellar white matter, but it did not correlate with α-synuclein load [79].

Cathepsin D has also been implicated in aspects of AD pathology in a dual manner. First, cathepsin D may act as a protease that cleaves the APP at the β-secretase site. It catalyzes this reaction with kinetics comparable to that of the “classical” β-secretase BACE1, but it is much more abundant in human brain than BACE1 (for review, see [104]. Second, cathepsin D was shown to hydrolyze Aβ peptides [105] and to regulate cerebral Aβ42/40 ratios via differential degradation of Aβ42 versus Aβ40. Genetic deletion of cathepsin D in mice resulted in 3- to 4-fold increases in Aβ42 and Aβ40, which was much higher than the augmentation after the deletion of other Aβ-degrading enzymes such as insulin-degrading enzyme and neprilysin [106]. Cathepsin D knock-out mice exhibited ≈30% increases in Aβ42/40 ratios, implicating a particular role of cathepsin D in the degradation of the more pathogenic Aβ42 variant. In addition, Aβ42 was shown to act as a competitive inhibitor of cathepsin D [106]. This may also result in inhibition of the clearance of neurotoxic tau species by cathepsin D within the lysosome [107]. Furthermore, a recent study indicates that cathepsin D may be a peripheral AD biomarker. Plasma cathepsin D levels quantified by immunoblotting and ELISA were found to be decreased in human subjects with amyloid plaque deposition compared to the control group [108]. In addition, the plasma cathepsin D level was negatively correlated with clinical dementia rating scale sum of boxes (CDR-SB) scores. Furthermore, an integrated multivariable logistic regression model using plasma cathepsin D levels allowed the discrimination of AD from non-AD [108].

## 5. α-Synuclein Cleavage by Matrix Metalloproteinase-3

Matrix metalloproteinases (MMPs) belong to the metzincin superfamily of metalloproteinases that also include a disintegrin and metalloproteinase (ADAM) and ADAM with thrombospondin motifs (ADAMTS) and are implicated in the proteolytical processing of α-synuclein (see [109] for a review). MMPs can be categorized according to their structure and substrate specificity into gelatinases (MMP-2 and MMP-9), stromelysins (MMP-3 and MMP-10), collagenases (MMP-1, MMP-8, and MMP-13), membrane-type MMPs and other MMPs [109]. MMPs are zinc- and calcium-dependent endopeptidases that share a high structural similarity and are synthesized by neurons, microglia and astrocytes [110,111]. They are implicated in the remodeling of the extracellular matrix, in particular under conditions of acute and chronic neurodegenerative disorders [112]. MMP expression can be induced by cytokines, nitric oxide, reactive oxygen species, and metabolites of the arachidonic pathway [113,114,115].

In addition, MMPs were identified as proteases catalyzing α-synuclein degradation [116,117]. Full-length α-synuclein constitutively secreted from human dopaminergic SK-N-BE cells was shown to be fragmented upon stimulation with NO donors [116]. This NO-induced proteolytical α-synuclein cleavage could be blocked by MMP inhibition. Subsequently, in vitro α-synuclein cleavage by a number of MMPs was followed by Western blot analysis, and the fragments generated were identified by mass spectrometry. This approach revealed most efficient α-synuclein cleavage by MMP-3, followed by MMP-14, MMP-2, MMP-1, and MMP-9 [116]. MMP-3 cleavage generated four major α-synuclein fragments, 1-54, 1-57, 1-78, and 1-79 (and their C-terminal counterparts) (Figure 1 and Table 1) as well as some minor fragments. Since most of the cleavage sites are located within the NAC domain, the authors also analyzed aggregation characteristics and neurotoxic profiles of maternal compared to MMP-3-cleaved α-synuclein fragments. Intriguingly, MMP-3-generated α-synuclein fragments displayed faster aggregation rates and higher neurotoxicity toward SK-N-BE cells but showed reduced fibril and more spherical granule formation, as revealed by electron microscopy [116]. It should be noted that the aggregation/neurotoxicity profiles reported represent that of mixed α-synuclein fragments derived by cleavage at different sites within the α-synuclein molecule.

Regarding the propagation of α-synuclein aggregates, prion-like seeding was reported for oligomer-like species but not for insoluble aggregates [118], and α-synuclein oligomers were shown to stabilize pre-existing defects in supported bilayers and to propagate membrane damage [119]. In living cells, the stabilization of α-synuclein oligomers resulted in increased cytotoxicity, which could be rescued by Hsp70 via the suppression of oligomer formation [120]. Thus, oligomer formation from MMP-3-generated α-synuclein fragments lacking parts of the NAC domain can lead to pathogenic protein assemblies. In the 6-OHDA rat PD model, increased MMP-3 expression in the SN was observed and discussed as being involved in the degeneration of dopaminergic neurons in this model [116].

Other groups confirmed and extended a role of MMPs in proteolytical α-synuclein degradation [117,121]. Using gel electrophoresis and mass spectrometry, the effects of MMP-1, MMP-3, and MMP-9 on α-synuclein cleavage were analyzed by Levin et al. [117]. This study confirmed the MMP-3 generated fragments identified by Sung et al. [116] but identified additional cleavage products. While the α-synuclein cleavage profiles for MMP-1, MMP-3, and MMP-9 were found to be distinct, one fragment (79–140) was generated by all the MMPs analyzed. The cleavages sites for MMP-1 and MMP-3 were preferentially localized within the vicinity of the NAC domain, and the resultant fragments showed increased de novo aggregation in vitro as analyzed by confocal single molecule spectroscopy. The increased α-synuclein aggregation could be blocked by MMP-3 inhibition. On the other hand, MMP-9 cleavage of α-synuclein did not enhance α-synuclein aggregation [117].

A pivotal role for MMP-3 in dopaminergic neuronal cell death was also reported in cell culture models and in experimental animals in vivo [121,122,123]. MMP-3 was demonstrated to be released from apoptotic neuronal rat PC12 cells and to activate microglia to express tumor necrosis factor-α, interleukin-6, interleukin-1β, and interleukin-1 receptor antagonist in vitro [122]. MMP-3 also caused the cell death of primary mouse mesencephalic dopaminergic neurons in mixed neuron–glia cultures in a NADPH-dependent manner. In the MPTP-induced PD mouse model, MMP-3 knock-out almost completely prevented dopaminergic neurodegeneration in SN and rescued concentrations of dopamine and its metabolites in striatum [121]. In addition, the co-localization of MMP-3 and α-synuclein was demonstrated in Lewy bodies in PD brain, and the co-overexpression of MMP-3 with α-synuclein resulted in an increase in protofibril-like small aggregates [123].

Interestingly, some MMPs including MMP-2, -9, -14, -16, and -24 have been implicated in proteolytical APP processing, giving rise to both amyloidogenic and non-amyloidogenic fragments (for a review, see [109]). Moreover, MMP-2, -3, -7, -9, and -14 were also shown to be involved in Aβ degradation [109]. In particular, the clioquinol-induced expression of MMP-3 resulted in instant degradation of Aβ secreted into the medium of cultured cells [124], and MMP-2 and -9 may contribute to extracellular brain Aβ clearance by promoting Aβ catabolism [125].

## 6. Conclusions

There are a number of proteolytical degradation systems for α-synuclein. Importantly, α-synuclein processing may occur at different intracellular compartments as well as extracellularly. Furthermore, the proteases capable of cleaving α-synuclein display distinct characteristics with regard to the processing of wild-type versus mutant α-synuclein and specificity for soluble versus aggregated α-synuclein. Interestingly, the specific cleavage sites for a given protease cluster at defined regions of the α-synuclein molecule. Depending on the specific cleavage, the proteases reviewed may affect α-synuclein aggregation propensity and neurotoxic profiles. Thus, the regulation of protease expression and/or modulation of its enzymatic activity does affect physiological and pathological outcomes of α-synuclein degradation. Intriguingly, the very same proteases implicated in α-synuclein degradation also play a role in the processing of the APP and/or degradation of Aβ peptides in AD. Thus, there might be common mechanisms of pathological protein processing shared between different clinical entities such as PD and AD. The clinical relevance of the proteases discussed is also emphasized by their potential usage as peripheral diagnostic markers and therapeutic targets.

## Figures and Tables

**Table 1:** α-Synuclein cleavage sites, localization, and references to proteases.

Enzyme	α-Synuclein Fragments		Primary Localization	Reference
	N-Terminal	C-Terminal		
Trypsin	1–21	22–140		Qin et al. [47]
	1–23	24–140		
	1–32	33–140		
MMP-3	1–54	55–140	Extracellular	Sung et al. [116] Levin et al. [117] Choi et al. [123]
	1–57	58–140		
	1–78	79–140		
	1–79	80–140		
Calpain-1	1–57	58–140	Cytosol	Mishizen-Eberz et al. [86]
	1–73	74–140		
	1–75	76–140		
	1–83	84–140		
	1–61	62–140	Cytosol	Shinkai-Ouchi et al. [89]
Cathepsin D	Syn10		Lysosome	Takahashi et al. [99]
	Syn12			
Neurosin	1–80	81–140	Cytosol and Extracellular	Kasai et al. [73]
	1–97	98–140		
	1–114	115–140		
	1–121	122–140		
